# Isoquercetin as an Adjunct Therapy in Patients With Kidney Cancer Receiving First-Line Sunitinib (QUASAR): Results of a Phase I Trial

**DOI:** 10.3389/fphar.2018.00189

**Published:** 2018-03-16

**Authors:** Carlo Buonerba, Pietro De Placido, Dario Bruzzese, Martina Pagliuca, Paola Ungaro, Davide Bosso, Dario Ribera, Simona Iaccarino, Luca Scafuri, Antonietta Liotti, Valeria Romeo, Michela Izzo, Francesco Perri, Beniamino Casale, Giuseppe Grimaldi, Francesca Vitrone, Arturo Brunetti, Daniela Terracciano, Alfredo Marinelli, Sabino De Placido, Giuseppe Di Lorenzo

**Affiliations:** ^1^Medical Oncology Division, Department of Clinical Medicine and Surgery, University Federico II of Naples, Naples, Italy; ^2^Istituto Zooprofilattico Sperimentale del Mezzogiorno, Portici, Italy; ^3^Department of Public Health, Federico II University of Naples, Naples, Italy; ^4^Institute of Experimental Endocrinology and Oncology (IEOS-CNR) “G. Salvatore”, Naples, Italy; ^5^Department of Translational Medical Sciences, University “Federico II”, Naples, Italy; ^6^Department of Advanced Biomedical Sciences, University Federico II of Naples, Naples, Italy; ^7^Medical Oncology Unit, POC SS Annunziata Taranto, Taranto, Italy; ^8^Dipartimento di Pneumologia e Tisiologia, Day Hospital Pneumologia e Pneumoncologico, AORN Vincenzo Monaldi, Naples, Italy; ^9^U.O. Medicina-Oncoematologia Ospedale Umberto I, Nocera Inferiore, Italy; ^10^IRCCS Istituto Neurologico Mediterraneo Neuromed, Pozzilli (IS), Italy

**Keywords:** sunitinib, isoquercetin, AMP-activated protein kinases, kidney cancer, phase I trials

## Abstract

Sunitinib is the most commonly prescribed drug for advanced renal cell carcinoma in the first-line setting and has been associated with multiple adverse events related to its on–and off–target effects, including hand and foot syndrome and fatigue. It was hypothesized that sunitinib-induced fatigue may be related to off target inhibition of the AMPK enzyme, which results in impairment of energy-producing processes at a systemic level. Quercetin is a naturally occurring flavonol with established AMPK-stimulating activity. While clinical use of quercetin is limited by its poor bio-availability, quercetin-3-O-β-d-glucopyranoside, that is isoquercetin, has an improved pharmacokinetic profile. On the grounds of the *in vitro* stimulatory activity with respect to AMPk, we hypothesized that oral isoquercetin could improve fatigue in kidney cancer patients receiving sunitinib. Given the lack of data on the safety of isoquercetin given concomitantly with sunitinib, we conducted a phase I trial to assess the safety of GMP manufactured isoquercetin given at two dose levels (450 and 900 mg a day). In the 12-patient study cohort included in this study, isoquercetin was administered concomitantly with 50 mg sunitinib for a median 81 days (IQR, 75.5, 86.5). None of the 12 patients required isoquercetin suspension or isoquercetin dose reduction because of adverse events. No abnormalities in ECG, heart or lower limbs doppler ultrasound were detected. A statistically significant improvement was reported for the FACIT fatigue score (6.8 points; 95% CI: 2.8–10.8; *p* = 0.002) and for the FACIT Adverse Events score (18.9 points; 95% CI: 9.1–28.8; *p* < 0.001) after isoquercetin consumption vs. baseline. In this phase I trial, isoquercetin was remarkably safe, with a preliminary signal of activity in terms of improvement of sunitinib adverse events.

## Background

In 2017, 63,990 people are estimated to have been diagnosed with renal cell carcinoma and 14,400 people are estimated to have died of the disease in the US (Siegel et al., 2017). Although metastatic disease is virtually incurable, prognosis has remarkably improved over the last decade thanks to the use of anti-VEGF agents (Buonerba et al., 2015), such as tyrosine kinase inhibitors sunitinib and pazopanib (Di Lorenzo et al., 2010). Sunitinib exerts its antineoplastic activity by inhibiting a number of molecular targets, which include platelet-derived growth factor receptors (PDGFRα and PDGFRβ), vascular endothelial growth factor receptors (VEGFR1, VEGFR2, and VEGFR3), stem cell factor receptor (KIT), Fms-like tyrosine kinase-3 (FLT3), colony stimulating factor receptor (CSF-1R), and the glial cell-line derived neurotrophic factor receptor (RET) (Papaetis and Syrigos, 2009). Sunitinib has been the most commonly prescribed first-line therapeutic agent over the last decade, although recent findings suggest alternative immunotherapy or targeted treatments may be more effective, especially in patients at poor prognosis (Powles et al., 2018). Sunitinib has been associated with multiple adverse events related to its on- and off-target effects, including hypertension, hypothyroidism, hand and foot syndrome, and fatigue (Di Lorenzo et al., 2011). Severity and incidence of some adverse events have been associated with certain gene polymorphisms, such as IL-8 and IL-13 and CYP3A5 (Garcia-Donas et al., 2011; Diekstra et al., 2015). In particular, fatigue has been reported in approximately half of unselected patients treated with sunitinib, with about one in 10 patients experiencing drug-related fatigue limiting self-care activities of daily living (Di Lorenzo et al., 2011). With respect to other tyrosine kinases inhibitors approved for the treatment of renal cell carcinoma, such as pazopanib and sorafenib, sunitinib is associated with increased rates of all- and high grade fatigue (Santoni et al., 2015). From a biological point of view, cancer-related fatigue has been associated with multiple putative mechanisms, including increased systemic inflammation levels and impaired mitochondrial function (Saligan et al., 2015). It was hypothesized that sunitinib-induced fatigue may be related to decreased expression of the GLUT transporters with decreased glucose intra-cellular uptake and off target inhibition of the AMPK enzyme, which results in impairment of catabolic, energy-producing processes, such as glycolysis and lipid oxidation at a systemic level (Aparicio et al., 2011). Quercetin is a naturally occurring flavonol characterized by a phenyl benzo(y)pyrone-derived structure, with putative antioxidative, anticancer, anti-inflammatory, and antidiabetic effects of potential clinical interest (Kawabata et al., 2015), and has been included among the flavonols with AMPK-stimulating properties (Russo et al., 2014). While clinical use of quercetin is limited by its poor bio-availability (Kawabata et al., 2015), quercetin-3-O-β-d-glucopyranoside, that is isoquercetin, has an improved pharmacokinetic profile (Stopa et al., 2017) and is efficiently converted *in vivo* into quercetin (Stopa et al., 2017), with peak concentration of quercetin of 9.2 μM after ingestion of 1,000 mg isoquercetin. On the grounds of *in vivo* isoquercetin conversion into quercetin, of quercetin *in vitro* stimulatory activity with respect to AMPk and GLUT transporters (Eid et al., 2010), as well as on its *in vivo* stimulatory activity on mitochondrial biogenesis (Lin et al., 2014), we hypothesized that a pharmacological intervention based on oral isoquercetin could improve fatigue in patients receiving sunitinib for advanced kidney cancer.

The QUASAR trial (Isoquercetin as an adjunct therapy in patients with kidney cancer receiving first-line sunitinib) is a phase I/IIb trial designed to test the safety of the combined use of sunitinib plus isoquercetin (phase I) and the anti-fatigue effect of isoquercetin vs. placebo (phase IIb) in a population of kidney cancer patients. We here present the findings of the phase I part of the QUASAR trial, which tested the safety of two dose levels (450 and 900 mg) of isoquercetin administered concomitantly with standard doses of sunitinib.

## Patients and methods

### Patients

Patients who received no prior systemic therapy other than sunitinib (including interleukin-2, interferon-α, chemotherapy, bevacizumab, mTOR inhibitor sorafenib, or other VEGF tyrosine kinasis inhibitor) for advanced or metastatic renal cell carcinoma with locally advanced (defined as disease not amenable to curative surgery or radiation therapy) or metastatic renal cell carcinoma of any histology (equivalent to Stage IV RCC according to AJCC staging) for whom treatment with sunitinib is either planned or ongoing were eligible to participate in the QUASAR trial. Only patients with a performance status of 0 or 1 were eligible. Patients who had been reported not to tolerate continuous 50 mg sunitinib or had had any major uncontrolled medical condition in the past 6 months were not eligible for the trial. All patients gave their written Ethics committee- approved informed consent prior to participating in the trial. The QUASAR trial was approved by all the competent authorities (Local Ethics Committee, Istituto Superiore di Sanità and AIFA) (ClinicalTrials.gov Identifier: NCT02446795).

### Study intervention

Isoquercetin (IQC-950AN) 225 mg plus ascorbic acid 55.8 mg and nicotinic acid 4.5 mg were administered twice a day without interruption in the first 6-patient cohort enrolled. Isoquercetin (IQC-950AN) 450 mg plus ascorbic acid 111.6 mg and nicotinic acid 9 mg were administered twice a day without interruption in the second six-patient cohort enrolled. Isoquercetin was delivered concomitantly with sunitinib for two (4:2) or four (2:1) sunitinib cycles. Sunitinib had to be initiated at the daily dose of 50 mg according to the schedule used at the time of study inclusion.

### Study assessments

Five study visits were scheduled: at screening, that is, on the last day sunitinib was administered during the last sunitinib cycle before inclusion; visit 1, that is, on the first day sunitinib was administered during the first sunitinib cycle after inclusion; visit 2, that is, on the last day sunitinib was administered during the first sunitinib cycle after inclusion; visit 3, that is on the first day sunitinib is administered during the second (in case of 4:2 schedule) or the fourth (in case 2:1 schedule) sunitinib cycle after inclusion; visit 4, that is on the last day sunitinib was administered during the second (in case of 4:2 schedule) or the fourth (in case 2:1 schedule) sunitinib cycle after inclusion.

Full blood count and blood chemistry, history and physical examination were performed, and questionnaires were administered at all study visits. The following questionnaires were used: (a) Fatigue–FACIT -Fatigue Questionnaire; (b) Quality of life—FACT-G; (c) Depression—PHQ-9 Patient Depression Questionnaire; (d) Anxiety—Generalized Anxiety Disorder (GAD-7); (e) Insomnia–Insomnia Symptoms questionnaire (ISQ); (f) Toxicity of anti-angiogenesis therapy- FACT-AntiA questionnaire.

Lower limbs venous ultrasonography, heart ultrasound, and 12-lead ECG were performed, and blood samples were collected for the translational study at baseline and at visit 4.

### Safety assessment

Toxicities associated with the combination of sunitinib and isoquercetin were evaluated throughout the study treatment and graded according to the NCI Common Toxicity Criteria 4.0.

The dose limiting toxicities (DLTs) considered for oral isoquercetin administered concomitantly with sunitinib were: Grade 2 or worse renal insufficiency; Grade 3-4 nausea; Grade 3-4 vomiting; Grade 3-4 diarrhea; Any serious adverse event related to concomitant administration of isoquercetin + sunitinib.

### Study procedures and design

In the phase I part of the study, we chose a 6 + 6 study design, rather than a 3 + 3 design, in order to assess the potential adverse events of isoquercetin in a larger patient sample (12 vs. 6). In fact, a classic 3 + 3 design would enroll only 6 patients, should no dose-limiting toxicites be reported–the most likely scenario given the low expected isoquercetin toxicity. The first six-patient cohort received 50 mg daily sunitinib + 450 mg daily isoquercetin. If no patient experienced DLTs until the completion of the study, then an additional six-patient cohort would be recruited to receive 900 mg daily isoquercetin. If one of the six-patient cohort treated with 450 mg daily experienced a DLT until the completion of the study, then the 450 mg dose of isoquercetin would be the MTD for the phase II trial. If more than one of the six patients planned to be recruited for the 450 mg dose level reported any DLT, then isoquercetin administration would be halted in all patients and the protocol would be amended to study lower doses of isoquercetin. If more than one patient treated at the 900 mg dose level reported any DLTs then the 450 mg would be the MTD. If not more than 1 patient experienced DLTs until the completion of the study in the six-patient cohort treated with 900 mg a day, then the 900 mg dose level would be the MTD of isoquercetin. The MTD will be used for the phase II part of the QUASAR trial. Patients experiencing toxicities regarded to be associated with isoquercetin that were not dose-limiting could be retreated at the same dose level upon full recovery. No pharmacokinetic or biomarker assessments were conducted in the phase I part of the QUASAR trial.

After study treatment was completed, patients would continue or discontinue sunitinib at the treating physician's discretion.

### Statistical analysis

All statistical analyses were performed using R (version 3.4.1; The R Foundation for Statistical Computing, Vienna, Austria).

Data were described using standard descriptive statistics. Categorical factors were summarized using counts and percentages while numerical variable were synthetized using mean ± standard deviation or median [25th; 75th percentile] in case of skewed distribution. Within-group comparisons were accordingly based either on the paired Student *t-*test or on the Wilcoxon test for related samples.

Longitudinal trajectories of efficacy outcomes in the whole cohort of patients were modeled using linear mixed models (LMMs) with time treated as categorical factor. Results of LMMs were reported as estimated difference from baseline with the corresponding 95% Confidence Intervals (95% C.I.). All test were two sided and *p* < 0.05 were considered statistically significant.

## Results

### Patients' characteristics

Twelve patients with clear cell renal carcinoma were included in this phase I study. Median age of the study population was 63.3 years (48; 83). Eleven patients were males and eleven had a performance status of 1, while one patient was a female and one had a performance status of 0. Eleven patients had metastatic disease, with lung, bone, adrenal, lymph-node, liver metastases being reported in 6, 4, 3, 3, and 1 patients, respectively. All patients had received sunitinib for advanced disease for a median of 13.9 months (IQR, 4.43–45.3). Median time from diagnosis to sunitinib initiation was 12 months (2; 46.5). All patients gave written informed consent to participate in the trial. Patients' characteristics are detailed in Table 1.

**Table 1:** Patients' characteristics.

	**Number of patients (%)**
**GENDER**	
Males	11 (91.7)
Female	1 (8.3)
**PERFORMANCE STATUS**	
0	1 (8.3)
1	11 (91.7)
Metastatic disease	11 (91.7)
Bone metastases	4 (33.3)
Adrenal metastases	3 (25)
Lymph nodes metastasis	3 (25)
Lung metastases	6 (50)
Liver metastases	1 (8.3)
Previous radical nephrectomy	10 (83.3)
Previous partial nephrectomy	1 (8.3)
Previous metastasectomy	4 (33.3)
Variable	Median (25th; 75th percentile)
Age; years	63.3 (48; 83)
Time from diagnosis to initiation of sunitinib; months	12 (2; 46.5)
Duration of previous sunitinib treatment; months	13.9 (4.43; 45.3)

### Treatment and safety

In the 12-patient study cohort, isoquercetin was administered concomitantly with 50 mg sunitinib for a median 81 days (IQR, 75.5, 86.5). Isoquercetin was remarkably safe, with none of the 12 patients requiring isoquercetin suspension or isoquercetin dose reduction because of adverse events. No abnormalities in ECG, heart or lower limbs doppler ultrasound were detected.

Sunitinib was administered at 50 mg according to the 4:2 schedule to 2 patients, while it was administered according to the 2:1 schedule in 10 patients. One patient had to suspend sunitinib for 20 days because of grade 2 diarrhea, while sunitinib dose was reduced from 50 to 37.5 mg in a 75-year old patient because of persistent grade 1 hypertension despite multi-drug anti-hypertensive therapy. There were no significant changes in laboratory parameters (see Table 2). No dose-limiting toxicities were reported either in the 450 mg or in the 900 mg isoquercetin cohort. All adverse events are reported in Table 3. We judged two events to be associated with isoquercetin consumption, with one patient reporting a single grade 1 flushing episode lasting <5 min after consuming isoquercetin and one patient reporting one episode of grade 1 flatulence after isoquercetin ingestion, which resolved spontaneously after <1 week. Concomitant medications are reported in Table 4. No clinically relevant drug-drug interaction was reported during study treatment at either dose level.

**Table 2:** Variations of laboratory parameters before and after isoquercetin in patients on sunitinib –^*^; ° Wilcoxon test for paired samples.

**Laboratory test—Unit of measure**	**Visit 1 Mean ± Standard deviation**	**Visit 4 Mean ± Standard deviation**	***P*-value *T-*test for paired sample**
Hemoglobin (g/dl)	13.8 ± 1.7	13.7 ± 2	0.876
Neutrophils (per mm3)	2.8 ± 1	2.5 ± 0.5	0.457
Lymphocytes (per mm3)	1.7 ± 0.4	1.7 ± 0.6	0.898
Platelet (per mm3)	233.1 ± 83.1	241.2 ± 79.2	0.750
FT3 pg/ml	2.7 ± 0.6	2.8 ± 0.5	0.807
Creatinin (mg/dL)	1.3 ± 0.2	1.4 ± 0.4	0.526
Total bilirubin (mg/dl)	0.7 ± 0.4	0.6 ± 0.3	0.712
SODIUM (mEq/L)	139.5 ± 4.1	139.6 ± 4	0.913
POTASSIUM (mEq/L)	4.6 ± 0.4	4.4 ± 0.4	0.142
CHLORIDE (mEq/L)	102.7 ± 1.6	103.2 ± 3.5	0.749
	**Visit 1 Median (25th; 75th percentile)**	**Visit 4 Median (25th; 75th percentile)**	***P*****-value Wilcoxon test for paired samples**
GGT (IU/L)	33 (19; 50.8)	26 (18; 54)	0.172
TSH (mIU/L)	3.9 (1.3; 12.6)	4 (1.5; 12.4)	0.859
Glycemia (mg/dL)	97.5 (86.8; 117.3)	85 (76; 104.5)	0.093
AST (IU/L)	23.5 (20.5; 41)	27 (22; 34)	0.444
ALT (IU/L)	27.5 (15.3; 34.3)	23 (18; 38)	0.964

**Table 3:** Adverse events reported during study treatment.

**Adverse event**	**Patients #*n* (%)**	**Possibly related to isoquercetin?**
**DIARRHEA**		
Grade 1	3	No
Grade 2	3	
**HAND AND FOOT SYNDROME**		
Grade 1	2	No
Grade 2	3	
**FATIGUE**		
Grade 1	7	No
Grade 2	1	
**SERUM CREATININE ELEVATION**		
Grade 1	1	No
**HYPERTENSION**		
Grade 1	4	No
**DYSGEUSIA**		
Grade 1	2	No
**OCULAR PAIN**		
Grade 1	1	No
**PERIPHERAL NEUROPATHY**		
Grade 1	1	No
**FLATULENCE**		
Grade 1	1	Yes
**FLUSHING**		
Grade 1	1	Yes

**Table 4:** Concomitant medications during study treatment.

**Concomitant drug**	**Patients consuming the drug (*n*)**
OMEPRAZOLO	4
LEVOTIROXINA	5
AMLODIPINA	5
ESOMEPRAZOLO	3
LANSOPRAZOLO	1
OLMESARTAN	2
ZOFENOPRIL	1
IDROCLORTIAZIDE	3
GLICLAZIDE	1
PARACETAMOLO	2
CODEINA	1
PERINDROPIL	2
SITAGLIPTIN	1
METFORMINA	1
DENOSUMAB	2
LOSARTAN	2
RANITIDINA	1
NADROPARINA	1
NEBIVOLOLO	2
DOXAZOSINA	2
FENOFIBRATO	1
COLECALCIFEROLO	1
ACIDO ACETIL SALICILICO	1
LECARNEDIPINA	2
FUROSEMIDE	2
SILODOSINA	1
VALSARTAN	1
SIMVASTATINA	1
LIOTIRONINA	1
ALLOPURINOLO	1
INDAPAMIDE	1
ENOXAPARINA	1
CANDESARTAN	1
TORASEMIDE	1
PANTOPRAZOLO	1
RIVAROXABAR	1
TAPENTADOL	1
PREGABALIN	1
LOPERAMIDE	3
DICLOFENAC	1
CEFTRIAXONE	1
PARNAPARINA	1
WARFARIN	1

## Efficacy outcomes

**Figure 1:**
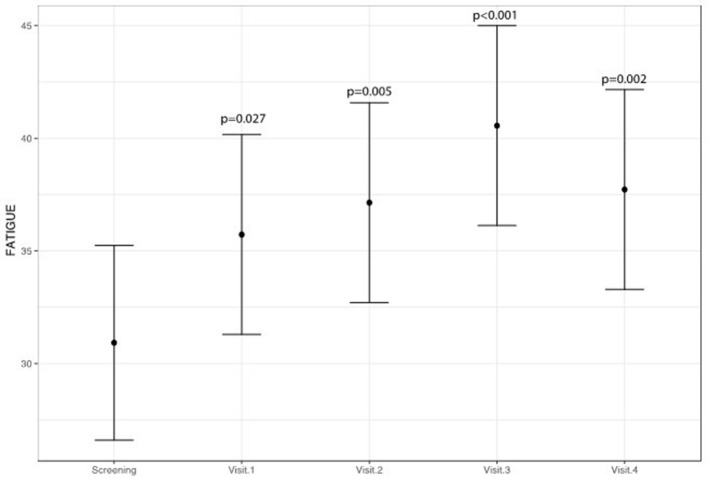
Variation of fatigue levels (higher scores indicate less fatigue).

**Figure 2:**
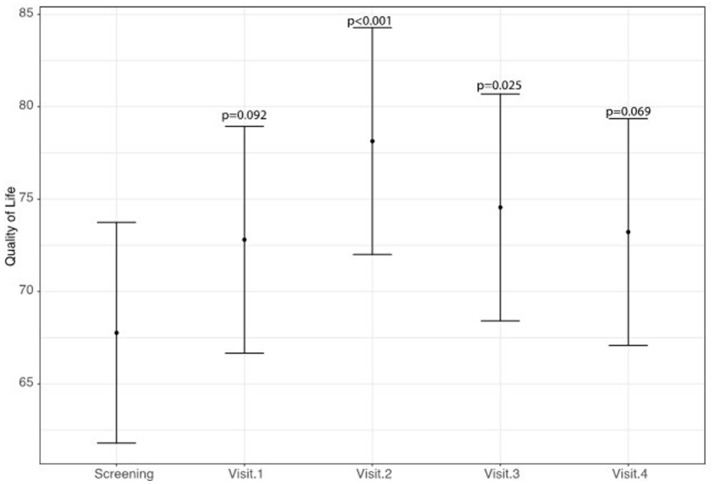
Variation of quality of life levels (higher scores indicate improved quality of life).

**Figure 3:**
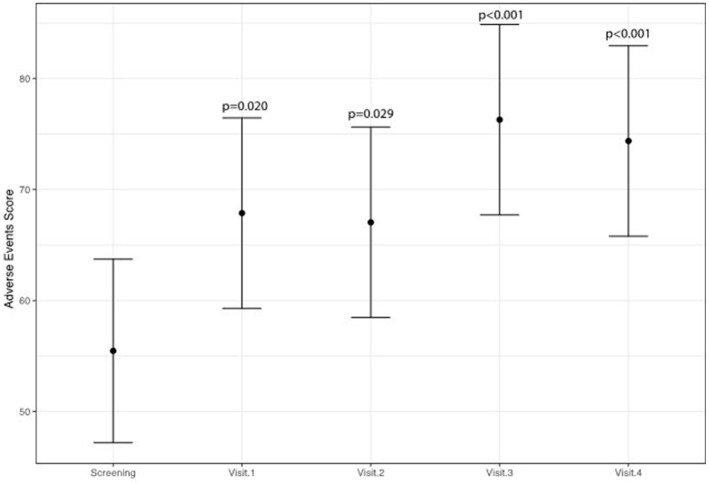
Variation of adverse events of anti-angiogenetic treatment levels (higher scores indicate less adverse events).

A statistically significant improvement at visit 4 vs. screening was reported for the fatigue score (6.8 points; 95% CI: 2.8–10.8; *p* = 0.002) and for the Adverse Events score (18.9 points; 95% CI: 9.1–28.8; *p* < 0.001). A borderline significant improvement in quality of life score (5.5 points; 95% CI: −0.1 to 11.1; *p* = 0.069) was also obtained (see Figures 1–3 and Table 5). Median scores obtained in single questions of the Adverse Events questionnaire are reported in Table 6. Clinically and statistically significant improvements were reported in questions regarding hand-and-foot syndrome (“The skin on my hands hurts”; “Hand pain or tenderness interferes with my daily activities”; “I am bothered by a skin rash”) and dysgeusia (“I am bothered by a change in the way food tastes”; “I am bothered by dry mouth”).

**Table 5:** Longitudinal variations of primary and secondary end points.

	**Quality of life score**	**Difference from baseline [95% C.I.]**
Screening	67.8 ± 9.4	–
Visit 1	73.6 ± 11.9	5 [−0.5 to 10.7] *p* = 0.092
Visit 2	78.9 ± 11.1	10.4 [4.8 to 16] *p* < 0.001
Visit 3	75.3 ± 9.2	6.8 [1.2 to 12.5] *p* = 0.025
Visit 4	74 ± 9.5	5.5 [−0.1 to 11.1] *p* = 0.069
	**Adverse events score**	**Difference from baseline [95% C.I.]**
Screening	55.5 ± 17.9	–
Visit 1	68.2 ± 17.1	12.4 [2.6 to 22.3] *p* = 0.02
Visit 2	67.4 ± 17.9	11.6 [1.8 to 21.5] *p* = 0.029
Visit 3	76.7 ± 8.2	20.8 [11 to 30.7] *p* < 0.001
Visit 4	74.8 ± 9.1	18.9 [9.1 to 28.8] *p* < 0.001
	**Fatigue**	**Difference from baseline [95% C.I.]**
Screening	30.9 ± 8.1	–
Visit 1	35.5 ± 7.2	4.8 [0.8 to 8.8] *p* = 0.027
Visit 2	36.9 ± 9	6.2 [2.2 to 10.2] *p* = 0.005
Visit 3	40.3 ± 4.9	9.6 [5.6 to 13.6] *p* < 0.001
Visit 4	37.5 ± 8.3	6.8 [2.8 to 10.8] *p* = 0.002
	**Functional well-being**	**Difference from baseline [95% C.I.]**
Screening	14.5 ± 5.4	–
Visit 1	15 ± 4.8	0.2 [−2.4 to 2.8] *p* = 0.873
Visit 2	15.2 ± 4.9	0.5 [−2.1 to 3.1] *p* = 0.731
Visit 3	15 ± 3.7	0.2 [−2.4 to 2.8] *p* = 0.873
Visit 4	13.3 ± 2.9	−1.4 [−4 to 1.2] *p* = 0.288
	**Emotional well-being**	**Difference from baseline [95% C.I.]**
Screening	17 ± 3.4	–
Visit 1	18.4 ± 3.7	1.3 [−0.4 to 3.1] *p* = 0.157
Visit 2	19.1 ± 2.5	2 [0.2 to 3.8] *p* = 0.036
Visit 3	18.7 ± 3.9	1.6 [−0.2 to 3.4] *p* = 0.094
Visit 4	18.7 ± 3.2	1.6 [−0.2 to 3.4] *p* = 0.094
	**Social/family well-being**	**Difference from baseline [95% C.I.]**
Screening	20.1 ± 4.1	–
Visit 1	20.1 ± 3	−0.3 [−2.8 to 2.3] *p* = 0.847
Visit 2	22 ± 4.8	1.7 [−0.9 to 4.3] *p* = 0.225
Visit 3	18.8 ± 4.6	−1.5 [−4.1 to 1.1] *p* = 0.267
Visit 4	19.5 ± 5.4	−0.8 [−3.4 to 1.8] *p* = 0.533
	**Physical well-being**	**Difference from baseline [95% C.I.]**
Screening	17.5 ± 4.6	–
Visit 1	20.1 ± 5.7	2.5 [0 to 5.1] *p* = 0.064
Visit 2	22.6 ± 3.7	5 [2.5 to 7.6] *p* < 0.001
Visit 3	22.8 ± 3.6	5.3 [2.7 to 7.8] *p* < 0.001
Visit 4	22.5 ± 2.4	4.9 [2.4 to 7.5] *p* < 0.001

*Changes from baseline, with the corresponding 95%C.I., were obtained using linear mixed models*.

**Table 6:** Individual questions of FACT-AntiA (Version 4) questionnaire in the entire study cohort.

**Question**	**Screening**	**End of follow up**	***p*-value**
I feel fatigued	2 [1.75; 2.25]	1 [0.75; 2]	0.016
I feel weak all over	1.5 [1; 2.25]	1 [1; 2]	0.258
My fatigue keeps me from doing the things I want to do	1 [0; 2]	1 [0; 1]	0.109
I am bothered by mouth sores or tenderness	2 [0.75; 2.25]	1 [0.75; 1.25]	0.063
Because of my mouth sores. Eating is difficult	1 [0; 2.25]	0 [0; 0.25]	0.063
The skin on my hands hurts	0.5 [0; 2.25]	0 [0; 0]	0.063
Hand pain or tenderness interferes with my daily activities	1 [0; 3.25]	0 [0; 1]	0.047
The skin on my feet hurts	0.5 [0; 3]	0 [0; 1]	0.031
Pain on the bottom of my feet interferes with my walking	1.5 [1; 3.25]	1 [0; 2.25]	0.297
I have diarrhea (diarrhoea)	2 [0; 3.25]	1 [0; 2]	0.188
I have to limit my activities because of diarrhea (diarrhoea)	1 [0; 2]	1 [0; 1]	0.344
I am bothered by a change in the way food tastes	1 [0; 1.25]	0.5 [0; 1]	0.156
I am bothered by dry mouth	3.5 [1.75; 4]	1 [1; 3]	0.047
I am bothered by headaches	3 [1.75; 3]	1 [0; 2]	0.004
I have pain in my joints	0 [0; 1.5]	0 [0; 0.25]	0.375
I am bothered by constipation	1 [0; 3]	1 [0; 2]	0.094
I am bothered by a skin rash	1.5 [0; 2.25]	0 [0; 1]	0.031
I am bothered by nosebleeds	2 [1.75; 3.25]	0.5 [0; 1.25]	0.007
I am bothered by hair loss	0 [0; 2.25]	0 [0; 0.25]	0.156
I am bothered by swelling in certain areas of my body	0 [0; 1]	0 [0; 0]	0.125
I have a loss of appetite	1 [0; 1.25]	0 [0; 1]	0.344
I have been short of breath	1 [0; 2.25]	0 [0; 0.25]	0.063
I have been vomiting	0 [0; 2]	0 [0; 0]	0.125

*Data are expressed as median [25th; 75th percentile]*.

A statistically significant improvement of 4.9 points for physical well-being was observed (95% CI: 2.4–7.5; *p* < 0.001) (Table 5). There were statistically significant improvements for the depression and anxiety scores, while no patients reported insomnia at screening or during study treatment (data not shown).

All patients had radiological stable disease at the end of study treatment. No deep venous thrombosis events were reported during study treatment.

## Discussion

According to the definition provided by the National Comprehensive Cancer Network guidelines (Berger et al., 2015), cancer-related fatigue is a distressing, persistent, subjective sense of physical, emotional, and/or cognitive tiredness or exhaustion related to cancer or cancer treatment that is not proportional to recent activity and interferes with the usual functioning. In kidney cancer patients treated with a biological agent, fatigue has been reported in up to 90% of patients, with a detrimental effect on quality of life (Goebell et al., 2016). In the particular setting of cancer patients receiving sunitinib, fatigue may be related to sunitinib-induced inhibition of the AMPK enzyme, which plays a key role in responding to ATP depletion via activation of multiple ATP-generating pathways (Aparicio et al., 2011). As reported by the NCCN guidelines, only a few pharmacological psychostimulant agents are included as potential effective interventions to relieve cancer-related fatigue. Although the use of nutritional supplements appears attractive for multiple reasons (e.g., because of their safety and patient compliance), none of those investigated in randomized controlled trials, including coenzyme Q10, American ginseng and acetylcarnitine, have proven to be more effective than placebo (Cruciani et al., 2012; Lesser et al., 2013; Yennurajalingam et al., 2017)- also because of a clinically significant placebo effect.

The biological rationale of administering isoquercetin to relieve fatigue in a population of sunitinib-treated patients is based on the potential stimulatory effect of quercetin on the AMPK enzyme shown in preclinical models. In one *in vitro* study, cells of a mouse myoblast cell line were exposed to 50 or 100 μM quercetin, and showed increased phosphorylation of Acetyl-CoA carboxylase, an AMPK effector, independently on PKB stimulation (Eid et al., 2010). In another cellular model, adipocytes 3T3-L1 cells exposed to increasing concentrations of quercetin (10, 50, and 100 μM), showed dose-correlated increasing up-regulation of the levels of phosphorylated adenosine monophosphate-activated protein kinase (AMPK) and its substrate, acetyl-CoA carboxylase (Ahn et al., 2008). A stimulatory effect on the AMPK activity was also observed in a mouse model of high-cholesterol-fed old mice consuming quercetin (Lu et al., 2010), while the quercetin derivative pentamethylquercetin increased both AMPK and GLUT4 activity in obese mice (Shen et al., 2012). One study examined the effects of 7 days of quercetin feedings in mice on endurance exercise tolerance. Mice were randomly assigned to one of the following three treatment groups: placebo, 12.5 or 25 mg/kg quercetin. Some mice also underwent a treadmill performance run to fatigue or were placed in voluntary activity wheel cages, and their voluntary activity (distance, time, and peak speed) was recorded. Quercetin increased mRNA expression of markers of mitochondrial biogenesis, that is PGC-1alpha and SIRT1 (*P* < 0.05), mtDNA (*P* < 0.05), and cytochrome c concentration (*P* < 0.05), and increased both maximal endurance capacity (*P* < 0.05) and voluntary wheel-running activity (*P* < 0.05; Davis et al., 2009). As far as clinical evidence is concerned, one meta-analysis identified eleven studies providing data on 254 human subjects. Across all studies, subject presupplementation VO(2max) ranged from 41 to 64 mL·kg^−1^·min^−1^ (median = 46), and median treatment duration was 11 days with a median dosage of 1,000 mg per day. Effect sizes were computed as the standardized mean difference, and meta-analyses were completed using a random-effects model. The effect size computed for all studies combining VO(2max) and endurance performance measures indicated a statistically significant, yet trivial-to-small in magnitude, effect favoring quercetin over placebo (effect size = 0.15, *P* = 0.021, 95% confidence interval = 0.02–0.27; Kressler et al., 2011).

Our study is the first published phase I trial that tested isoquercetin not as a nutritional supplement or an extract but as a GMP-manufactured pharmacological agent. Isoquercetin was administered in combination with low doses of vitamin B3 and C at the manufacturer's request to use vitamin C and B3 as stabilizers to prevent isoquercetin oxidation. As in both the 450 and 900 mg arms the total doses of vitamin C and B3 delivered were, respectively, 100–200 and 9–18 mg, which were largely within safety limits and below or near doses commonly used for supplementation (Hageman et al., 1998; Hemila and Chalker, 2013), we considered that co-administration of B3 and C was highly unlikely to cause adverse events and consented to use the manufacturer's formula. Importantly, we decided to use vitamin C and B3, rather than a completely inert placebo, in the control arm of the phase II part of the trial, in order to exclude that these vitamins could play a significant role in any anti-fatigue effect seen. In the only phase I study conducted on quercetin, quercetin was administered intravenously up to the dose of 1,700 mg/m^2^ at 3-weekly or weekly intervals in a cohort of 51 patients with advanced cancer. Kidney failure, which could be partially prevented by intravenous hydration, was the only dose-limiting toxicity, and the authors concluded that the dose suggested for a phase II trial for intravenous use was 1,400 mg/m^2^ at 3-week or weekly intervals, although grade 3 renal toxicity was reported in one patient receiving 630 mg/m^2^ intravenous quercetin (Ferry et al., 1996). Quercetin is considered as a safe nutritional ingredient by the FDA up to the dose of 500 mg per serving^1^ Presently, clinical data about the safety of quercetin and isoquercetin are available from clinical trials using quercetin or its related compounds as nutritional supplements or extracts (Shoskes et al., 1999; Kiesewetter et al., 2000; Lozoya et al., 2002; Shanely et al., 2010). The Institutional Review Board at the Dana-Farber Institution and the FDA have recently approved two dose levels of isoquercetin, 500 and 1,000 mg, to be simultaneously tested in the phase IIb part of a large phase II/III trial for thrombosis prevention in cancer patients (ClinicalTrials.gov: NCT02195232). Our phase I study is the first to assess the potential interactions of GMP-manufactured isoquercetin with sunitinib in a selected population of kidney cancer patients. In our phase I trial, we found that up to 900 mg a day isoquercetin is remarkably safe, with no patient having to reduce or suspend isoquercetin, no clinically relevant drug-drug interaction, and only two single, short-timed episodes of grade 1 flatulence and grade 1 flushing judged to be possibly associated with isoquercetin. The adverse events reported overall are consistent with those known to be related to sunitinib (Di Lorenzo et al., 2011). We purposely enrolled a cohort of patients who had shown good tolerance to sunitinib to better identify potential and unexpected adverse events potentially associated with isoquercetin. Conversely, a statistically significant improvement of the score of fatigue and toxicity related to anti-VEGF agent score levels measured on the last day of sunitinib before starting isoquercetin vs. the last day of isoquercetin before stopping sunitinib was noted. In particular, we found a signal for reduced fatigue, hand-and-foot syndrome and dysgeusia associated with isoquercetin consumption. Although these findings are encouraging, the magnitude of the placebo effect cannot be estimated in this single-arm cohort, and randomized-controlled trials are required.

In conclusion, we found that isoquercetin coadministered with low oral doses of vitamin C and B3 had an excellent safety profile, patient compliance and encouraging activity in terms of improvement of fatigue, possibly, other adverse events (e.g., hand and foot syndrome) associated with sunitinib. Continuation of phase I/II QUASAR trial is warranted using isoquercetin 900 mg/day.

## Author contributions

GDL and CB: study conception and design, obtaining funds, protocol writing; GDL, CB, PDP, PU, AL, DT and FP: manuscript writing; DBr: statistics; PDP, MP, DBo, DR, SI, VR, MI, BC, GG, FV, LS: data collection; AB, DT, AM, SDP: supervision.

### Conflict of interest statement

The authors declare that the research was conducted in the absence of any commercial or financial relationships that could be construed as a potential conflict of interest.

## Footnotes

^1^https://www.accessdata.fda.gov/scripts/fdcc/index.cfm?set=GRASNotices&id=341.
